# Panel of three cytokines predicts occurrence of aGVHD after allo-HSCT: a retrospective study

**DOI:** 10.3389/fimmu.2025.1719287

**Published:** 2026-01-13

**Authors:** Lin Xia, Yue Wang, Shaping Deng, Yuqiu Qi, Zhuo Wang

**Affiliations:** 1 Department of Pharmacy, Shanghai Changhai Hospital, The First Affiliated Hospital of Naval Medical University, Shanghai, China; 2 School of Pharmacy, Jiangxi University of Chinese Medicine, Nanchang, China

**Keywords:** acute graft-versus-host-disease, allogeneic hematopoietic stem cell transplantation, IL-10, IL-5, IL-8

## Abstract

**Introduction:**

Many cytokines have been used as candidate biomarkers of acute graft-versus-host disease (aGVHD). Among these, the roles of interferon (IFN)-γ, tumor necrosis factor (TNF)-α, interleukin (IL)-6, IL-8, IL-10, IL-17A, and IL-1β in aGVHD remain debatable, whereas IL-2, IL-12P70, IL-4, IL-5, and IFN-α are key elements in the pathological process of aGVHD. This study aimed to verify whether these 12 cytokines could serve as potential biomarkers of aGVHD in patients who underwent allogeneic hematopoietic stem cell transplantation (allo-HSCT).

**Methods:**

In this retrospective study, 155 patients were stratified into control (non-aGVHD) and experimental (aGVHD) groups based on the occurrence of aGVHD. The association between cytokine levels and aGVHD occurrence was evaluated.

**Results:**

The expression levels of IL-5, IL-8, and IL-10 were significantly elevated in patients with aGVHD compared to those in patients without aGVHD. The results of multivariate analysis revealed that IL-5, IL-8, and IL-10 were independent risk variables for aGVHD. These three cytokines formed a composite biomarker panel with a good predictive ability for aGVHD. The panel remained an independent predictor in multivariable analysis (HR = 3.34, 95% CI: 1.66 - 6.69, P < 0.001). The composite biomarker panel demonstrated robust discriminative performance upon internal validation with 1000 bootstrap resamples.

**Discussion:**

The composite biomarker panel comprising IL-5, IL-8, and IL-10 may serve as an important biomarker for predicting aGVHD occurrence.

## 1 Introduction

Allogeneic hematopoietic stem cell transplantation (allo-HSCT) is an effective therapy for high-risk hematological malignancies, other life-threatening diseases, and genetic disorders (1, 2). However, the extensive clinical application of allo-HSCT is limited by the occurrence of acute graft-versus-host disease (aGVHD) (1, 3). aGVHD is a common immune complication of allo-HSCT and the most frequent cause of non-relapse mortality after transplantation (4–6). aGVHD is mainly caused by an immune response against recipient cells, which is triggered by activated donor immune cells. The complication typically occurs within 100 days of allo-HSCT, primarily affecting the skin, gastrointestinal tract, and liver (4, 7–9).

The diagnosis of aGVHD mainly relies on clinical features and pathological biopsies of the target organs, which lack specificity and clinical feasibility, respectively (7, 10). Consequently, many studies have focused on identifying aGVHD-related biological markers (11, 12). Cytokines are small-molecule proteins secreted by immune cells that play crucial roles in the regulation of immune and inflammatory responses. Pro-inflammatory and anti-inflammatory cytokines can provide specific indications of the occurrence and progression of aGVHD (13). Cytokines such as interleukin (IL)-6, IL-8, and IL-2 have been used as candidate biomarkers of aGVHD (11, 14, 15). Some studies have reported significant differences in IL-6 levels between patients who did and did not develop aGVHD (16–18). However, the results of subsequent studies did not corroborate these findings (10, 19, 20). Besides IL-6’s role, the roles of other cytokines, such as interferon (IFN)-γ, tumor necrosis factor (TNF)-α, IL-8, IL-10, IL-17A, and IL-1β, as biomarkers of aGVHD are unclear (10, 11, 13, 20–23). Apart from the aforementioned cytokines, the cytokines IL-2, IL-12P70, IL-4, IL-5, and IFN-α are core elements in the pathological process of aGVHD (13, 18).

The selected cytokines correspond to the core immunopathological processes of aGVHD. Initial tissue injury leads to a substantial release of cytokines, such as TNF-α, IL-1β, IL-6, and IL-8, which contribute to generating a proinflammatory milieu (24, 25). This environment promotes donor T-cell activation and polarization, driven by cytokines including IL-2, IL-12p70, IFN-γ, IL-17A, IL-4, and IL-5 (24, 25). In addition, IL-10 plays a pivotal immunoregulatory role (26), and IFN-α contributes in differentiating aGVHD from viral infection. Collectively, these 12 cytokines comprehensively cover key pathways in aGVHD immunobiology, spanning innate immunity, T-cell activation/polarization, and immunoregulation.

This study aimed to determine the levels of 12 cytokines in patients who underwent allo-HSCT and investigate the association between the concentrations of these cytokines and aGVHD occurrence in these patients. The results will help in establishing a cocktail of cytokines to predict the onset of aGVHD in patients who underwent allo-HSCT.

## 2 Materials and methods

### 2.1 Study population

This retrospective study included 155 patients who underwent allo-HSCT at the Shanghai Changhai Hospital between January 2022 and May 2025. The diagnosis of aGVHD was carried out according to the Mount Sinai Acute GVHD International Consortium (MAGIC) criteria (9, 27, 28). The control and experimental groups comprised the non-aGVHD and aGVHD groups, respectively. The study protocol was approved by the Ethics Committee of the Shanghai Changhai Hospital (CHEC 2025-404).

### 2.2 Conditioning and GVHD prophylaxis regimen

A modified busulfan/cyclophosphamide (BU/CY) regimen was used as the primary myeloablative conditioning regimen. The modified BU/CY regimen comprised high-dose cytarabine (3 g/m^2^/day, days -9 to -8), busulfan (3.2 mg/kg/day, days -7 to -5), cyclophosphamide (1.8 g/m^2^/day, days -4 to -3), and semustine (methyl-CCNU; 250 mg/m^2^/day, days -3). Furthermore, rabbit anti-thymocyte globulin was administered at a dosage of 2.5 mg/kg/day (days -4 to -1). All patients received a calcineurin inhibitor (tacrolimus or cyclosporine), mycophenolate mofetil, and a short course of methotrexate (15 mg/m^2^ on days +1; 10 mg/m^2^ on days +3 and +6) for aGVHD prophylaxis. The dosages of tacrolimus or cyclosporine were adjusted based on their blood concentrations, with target ranges of 5–15 µg/L and 150–250 µg/L, respectively.

### 2.3 Sample collection and cytokine measurement

The last blood sample collected from patients with aGVHD prior to diagnosis was compared with samples obtained from patients without aGVHD at equivalent time points after transplantation. The median time to the occurrence of aGVHD in the experimental group was 21.00 (interquartile range [IQR]: 17.00 - 29.00) days after transplantation. Samples from this group were drawn at a median of 1.00 (IQR: 1.00 - 3.00) day prior to diagnosis. Control samples (from patients without aGVHD) were collected at a matched median time of 20.00 (IQR: 19.00 - 21.00) days post-transplant. Cytokine levels in blood samples were measured using a double-antibody sandwich enzyme-linked immunosorbent assay kit (Human Cytokine Assay Kit; Genzyme Corporation, USA). The concentrations are reported in picograms per milliliter (pg/mL).

### 2.4 Statistical analysis

The R software was used for statistical analyses. Normally distributed continuous variables are presented as means ± standard deviations (SDs) and were compared using the t-test. Non-normal continuous variables are presented as medians with IQR and were compared using the Wilcoxon signed-rank test. Categorical variables are presented as numbers with percentages and were compared using the chi-square or Fisher’s exact test. The diagnostic values of the biomarkers were assessed using receiver operating characteristic (ROC) curves and the area under the curve (AUC) to establish the optimal cut-off values. The patients were subsequently categorized into high- and low-level groups based on their cut-off values. Only biomarkers demonstrating significant discriminatory power in the ROC analysis were further analyzed using Kaplan-Meier survival curves, and the cumulative incidence of aGVHD between the two groups was compared with the log-rank test. A univariate Cox regression analysis was performed to screen the cytokines. Subsequently, significant variables were incorporated into a multivariate Cox proportional hazards model. A logistic regression model was developed to predict aGVHD using the identified variables. Based on the resulting model coefficients, a composite risk score was calculated for each patient as a weighted linear combination of the cytokine levels. This composite score was then used in subsequent analyses as a single, continuous variable representing the risk of aGVHD. To evaluate the stability of the model’s performance, an internal bootstrap validation with 1,000 replicates was conducted. The AUC was calculated for each bootstrap sample, and the distribution of these 1,000 AUC values was examined. P values were adjusted for multiple comparisons using Benjamini–Hochberg false discovery rate correction. Differences between patients in the aGVHD and non-aGVHD groups were considered statistically significant at P < 0.05.

## 3 Results

### 3.1 Demographic characteristics

This study included 156 patients who underwent allo-HSCT. The study population consisted of 30 (19.23%) patients with acute lymphoblastic leukemia (ALL), 86 (55.13%) with acute myeloid leukemia (AML), six (3.85%) with mixed-phenotype acute leukemia (MPAL), seven (4.49%) with chronic myeloid leukemia (CML), 24 (15.38%) with myelodysplastic syndrome (MDS), and three (1.92%) with non-Hodgkin’s lymphoma (NHL). The aGVHD group comprised 64 (41.03%) patients, of whom 56.25% (36) were men, and the median age was 44.50 (IQR: 32.00 - 55.50) years. The non-aGVHD group comprised 92 (58.97%) patients, of whom 64.13% (59) were men, and the median age was 43.00 (IQR: 31.00 - 53.00) years. No significant differences were observed between the clinical characteristics of patients in the aGVHD and non-aGVHD groups, except for graft source, donor source, and human leukocyte antigen (HLA) matching ratios. HLA matching ratio was determined by the proportion of matched alleles at the HLA-A, -B, -C, and -DRB1 loci. The clinical characteristics of the patients are listed in **Table 1**.

**Table 1:** Baseline clinical and demographic characteristics of the study cohort.

Characteristic	non-aGVHD	aGVHD	P
N (%)	92 (58.97)	64 (41.03)	
Age (years)	43.00 (31.00 - 53.00)	44.50 (32.00 - 55.50)	0.573
Sex, N (%)			0.409
Male	59 (64.13)	36 (56.25)	
Female	33 (35.87)	28 (43.75)	
Sex Matching, N (%) (Donor → Recipient)			0.391
Male → Male	45 (48.91)	27 (42.19)	
Male → Female	22 (23.91)	24 (37.50)	
Female → Male	14 (15.22)	9 (14.06)	
Female → Female	8 (8.70)	4 (6.25)	
Donor Age (years)	32.00 (25.00 - 36.00)	30.00 (26.50 - 37.00)	0.751
Height (cm)	170.00 (162.00 - 175.00)	168.00 (160.75 - 174.00)	0.660
Weight (kg)	66.65 (57.55 - 73.90)	64.95 (56.85 - 71.02)	0.477
Diagnosis, N (%)			0.903
ALL	15 (16.30)	15 (23.44)	
AML	52 (56.52)	34 (53.12)	
MPAL	4 (4.35)	2 (3.12)	
CML	5 (5.43)	2 (3.12)	
MDS	14 (15.22)	10 (15.62)	
NHL	2 (2.17)	1 (1.56)	
Donor Source, N (%)			0.001
MRD	14 (15.22)	3 (4.69)	
MMRD	3 (3.26)	4 (6.25)	
Haplo	28 (30.43)	39 (60.94)	
MUD	26 (28.26)	9 (14.06)	
MMUD	21 (22.83)	9 (14.06)	
Graft Source, N (%)			0.008
Bone marrow	1 (1.09)	0 (0.00)	
Peripheral Blood Stem Cells	70 (76.09)	36 (56.25)	
Bone marrow + Peripheral Blood Stem Cells	21 (22.83)	28 (43.75)	
HLA Matching Ratio	0.90 (0.58 - 1.00)	0.67 (0.50 - 0.90)	< 0.001
ABO Mismatch, N (%)			0.621
None	41 (44.57)	35 (54.69)	
Minor	22 (23.91)	11 (17.19)	
Major	20 (21.74)	12 (18.75)	
Bidirectional	9 (9.78)	6 (9.38)	
Virus, N (%)			0.492
Positive	6 (6.52)	7 (10.94)	
Negative	86 (93.48)	57 (89.06)	
Bacteria, N (%)			1.000
Positive	6 (6.52)	4 (6.25)	
Negative	86 (93.48)	60 (93.75)	
Fungi, N (%)			0.167
Positive	0 (0.00)	2 (3.12)	
Negative	92 (100.00)	62 (96.88)	
Onset of aGVHD (days)	–	21.00 (17.00 - 29.00)	
Sample Collection Time point (days)	20.00 (19.00 - 21.00)	19.00 (14.00 - 25.00)	0.162
Granulocyte Engraftment Time (days)	13.00 (11.00 - 15.00)	12.00 (11.25 - 14.00)	0.526
Megakaryocyte Engraftment Time (days)	13.00 (12.00 - 15.00)	13.00 (12.00 - 16.50)	0.741
Hematopoietic Recovery Time (days)	19.00 (15.00 - 25.00)	16.00 (14.00 - 21.00)	0.080

Numerical variables were expressed as median (IQR), categorical variables were presented as numbers with percentages; HLA-matching ratio was determined by the proportion of matched alleles at the HLA-A, -B, -C, and -DRB1 loci; ABO mismatch: None, donor and recipient have the same ABO blood type; Minor, donor has antibodies against recipient ABO antigens; Major, recipient has antibodies against donor ABO antigens; Bidirectional, both major and minor incompatibility exist; Days indicated the post-transplantation period.

N, Number; ALL, Acute Lymphoblastic Leukemia; AML, Acute Myeloid Leukemia; MPAL, Mixed-Phenotype Acute Leukemia; CML, Chronic Myeloid Leukemia; MDS, Myelodysplastic Syndromes; NHL: Non-Hodgkin’s Lymphoma; MRD, Matched Related Donor; MMRD, Mismatched Related Donor; Haplo, Haploidentical Donor; MUD, Matched Unrelated Donor; MMUD, Mismatched Unrelated Donor; HLA, human leukocyte antigen.

### 3.2 Discovery of aGVHD biomarkers

**Figure 1:**
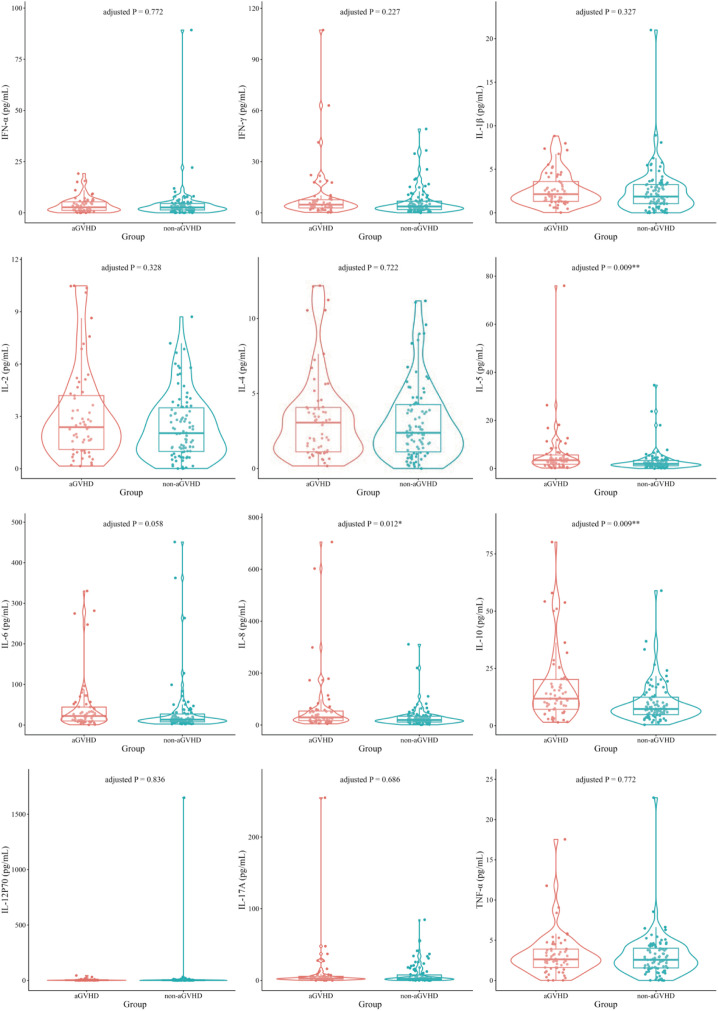
Comparison of cytokine levels between aGVHD and non-aGVHD patients. Violin plots with overlaid scatter plots show the concentrations of IFN-α, IFN-γ, IL-1β, IL-2, IL-4, IL-5,IL-6, IL-8, IL-10, IL-12P70, IL-17A, and TNF-α. Each point represents an individual patient. Box plot elements indicate the median and interquartile range. Statistical significance was determined by the MannWhitney U test. P values were adjusted for multiple comparisons using the Benjamini-Hochberg false discovery rate method (*adjusted P < 0.05; **adjusted P < 0.01; ***adjusted P < 0.001).

Samples were collected from 151 patients, of whom 61 developed aGVHD. Statistical analysis revealed that of the 12 cytokines, three cytokines differed between the two groups. The levels of IL-5, IL-8, and IL-10 in the aGVHD group were significantly higher than those in the non-aGVHD group (**Figure 1**; **Supplementary Table S1**). The ROC curve analysis (**Supplementary Figure S1**; **Supplementary Table S2**) revealed that IL-10 exhibited the optimal predictive ability for aGVHD (AUC: 0.662, 95% confidence interval [CI]: 0.566- 0.752; sensitivity, 70.49%; specificity, 62.22%; adjusted P < 0.001), followed by the predictive abilities of IL-5 (AUC: 0.652, 95%CI: 0.562 - 0.750; sensitivity: 63.93%; specificity: 65.56%; adjusted P = 0.006). The Kaplan-Meier survival analysis (**Supplementary Figure S2**) demonstrated that patients with high-level group of the two aforementioned cytokines had a significantly higher cumulative incidence of aGVHD compared to those with low-level group. This association was particularly strong for IL-10, IL-5 (adjusted P < 0.001).

**Figure 2:**
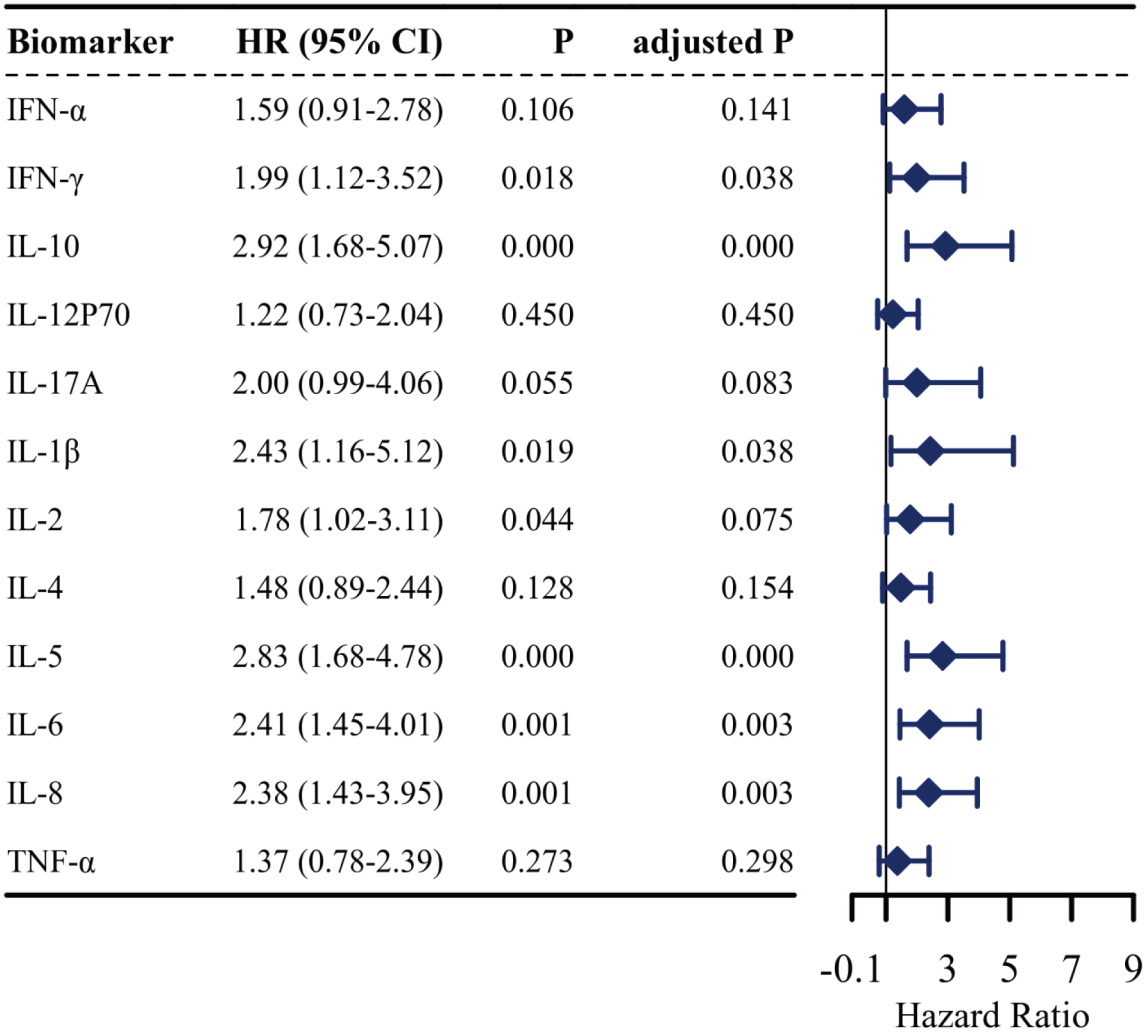
Univariate Cox regression analysis of potential risk factors for aGVHD. Forest plot shows the unadjusted hazard ratios (HR) and 95% confidence intervals (CI) for each variable. An HR greater than 1 indicates an increased risk of aGVHD. P values were adjusted for multiple comparisons using the Benjamini-Hochberg false discovery rate method.

**Figure 3:**
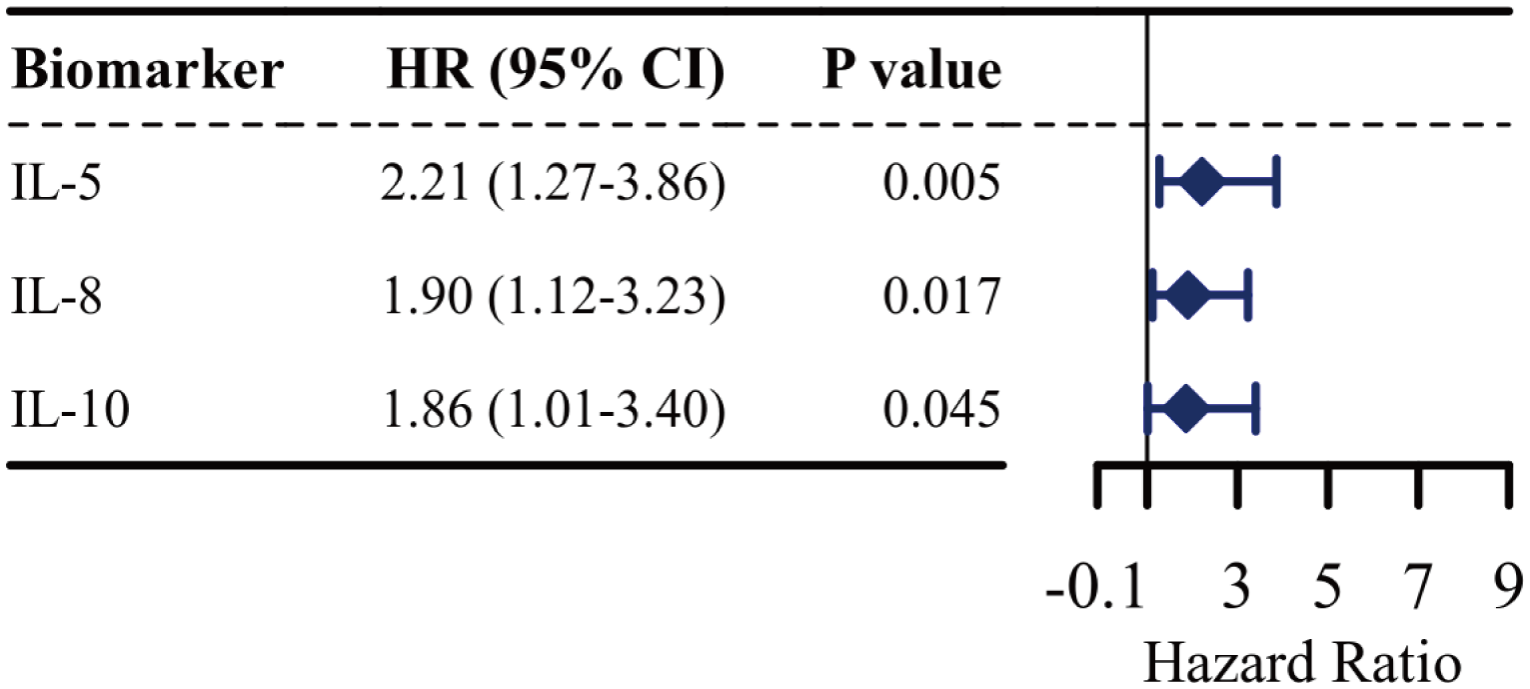
Multivariate Cox regression model for aGVHD. Forest plot shows the adjusted hazard ratios (HR) and 95% confidence intervals (CI) for each variable in the final model. The analysis was adjusted for IL-5, IL-8, and IL-10. An HR greater than 1 indicates increased risk of aGVHD.

To assess the effects of the 12 cytokines on the risk of aGVHD development, a univariate Cox regression model was developed. As shown in **Figure 2**, six cytokines other than IFN-α, IL-2, IL-4, IL-12P70, IL-17A, and TNF-α were associated with an increased risk of aGVHD. Furthermore, the multivariable analysis results revealed that IL-5 (hazard ratio [HR]: 2.21 95% CI: 1.27 - 3.86; P = 0.005), IL-8 (HR: 1.90, 95% CI: 1.12 - 3.23; P = 0.017), and IL-10 (HR: 1.86, 95% CI: 1.01 - 3.40; P = 0.045) were independent risk variables for aGVHD (**Figure 3**).

**Figure 4:**
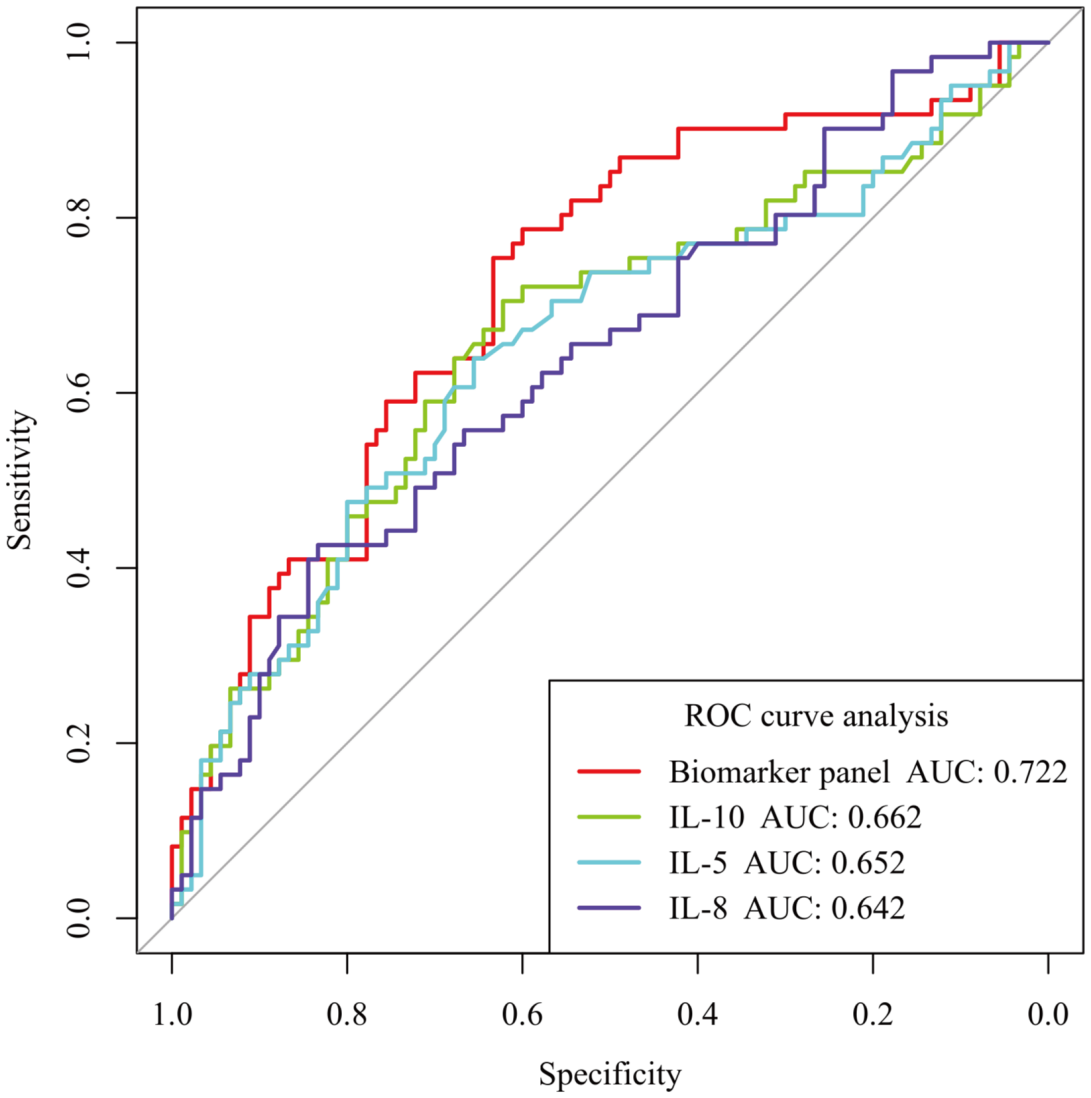
Comparison of the predictive performance for aGVHD between individual cytokines and a composite biomarker panel. Receiver operating characteristic (ROC) curves show that the composite biomarker panel (IL-5, IL-8, and IL-10) achieved a higher area under the curve (AUC = 0.722 [95% CI: 0.629 - 0.806]; sensitivity, 75.41%; specificity; 63.33%; adjusted P < 0.001) than any cytokine alone. Optimal cut-off values from ROC analysis are: biomarker panel risk score -0.681, IL-10 8.615 pg/mL, IL-5 2.600 pg/mL, and IL-8 36.820pg/mL.

The three screened biomarkers, IL-5, IL-8, and IL-10, were analyzed using a logistic regression model to construct a composite biomarker panel. The formula for the composite risk score is Y = 0.0649× IL-5 + 0.0052 × IL-8 + 0.0408× IL-10 - 1.3839. ROC curves were analyzed to assess the risk of aGVHD occurrence using the panel. The AUC for the panel was 0.722 [95% CI: 0.629 - 0.806], with a sensitivity of 75.41% and a specificity of 63.33% (adjusted P < 0.001). As shown in **Figure 4**, the AUC for the composite biomarker panel surpassed that of the single biomarker.

To determine whether the composite biomarker panel provided independent predictive ability beyond clinical risk factors, a multivariable Cox regression analysis was performed. The model included the composite risk score, along with age, sex, sex matching, height, weight, diagnosis, HLA ratio, granulocyte engraftment time, megakaryocyte engraftment time, hematopoietic recovery time, donor source, ABO mismatch, and Infection with bacteria, viruses, or fungi. As summarized in **Table 2**, a high-risk composite score remained significantly associated with an increased incidence of aGVHD (HR = 3.34, 95% CI: 1.66 -6.69, P < 0.001) after adjustment for these clinical variables. In contrast, none of the clinical factors were identified as independent predictors in the model. These results confirm that the composite biomarker panel is a strong and independent predictor of aGVHD risk.

**Table 2:** Multivariable analysis of factors associated with aGVHD risk.

Variable	HR (95% CI)	P
Age	1.00 (0.98 - 1.03)	0.906
Sex	0.52 (0.20 - 1.37)	0.188
Sex matching	1.08 (0.74 - 1.56)	0.699
Height	1.03 (0.98 - 1.08)	0.277
Weight	0.99 (0.96 - 1.02)	0.386
Diagnosis	0.89 (0.67 - 1.19)	0.424
HLA ratio	0.14 (0.00 - 6.33)	0.308
Granulocyte engraftment time	0.90 (0.73 - 1.12)	0.361
Megakaryocyte engraftment time	1.05 (0.92 - 1.20)	0.482
Hematopoietic recovery time	0.96 (0.90 - 1.02)	0.222
Donor source	0.92 (0.55 - 1.54)	0.755
ABO mismatch	0.95 (0.70 - 1.28)	0.715
Infection	1.07 (0.49 - 2.32)	0.872
Biomarker panel	3.34 (1.66 - 6.69)	<0.001

An HR greater than 1 indicates an increased risk of aGVHD. Infection includes bacteria, viruses, and fungi.

The formula for the composite biomarker panel risk score is Y = 0.0649× IL-5 + 0.0052 × IL-8 + 0.0408× IL-10 - 1.3839 (Cut-off value: -0.681).

HR, Hazard Ratio; CI, confidence interval.

### 3.3 Validation of aGVHD biomarkers

**Figure 5:**
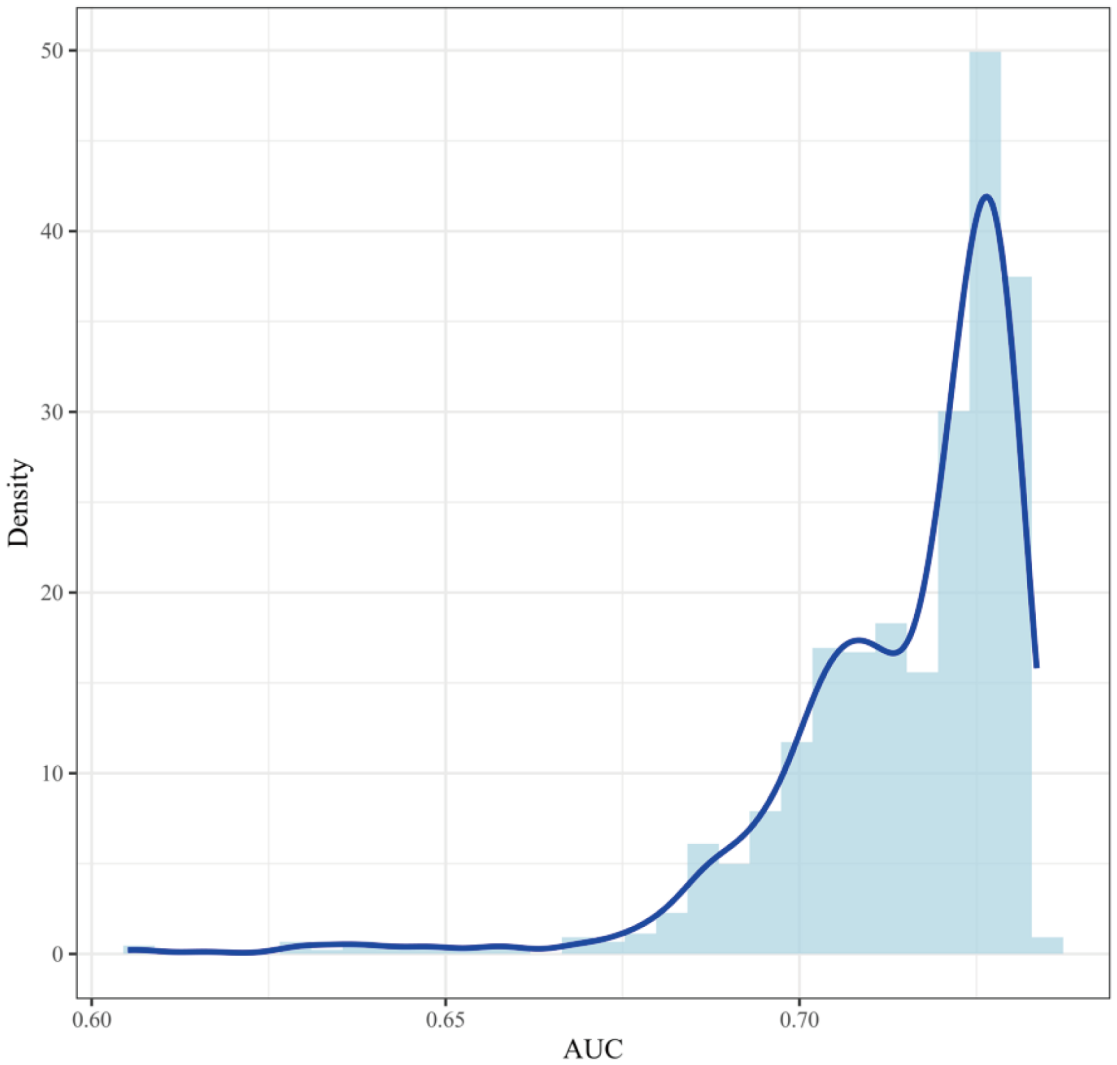
Internal bootstrap validation of the composite biomarker panel for predicting aGVHD. The histogram and density curve (blue) depict the distribution of the Area Under the Curve (AUC) generated from 1000 internal bootstrap replications, illustrating the robustness of the model’s discriminatory performance. The mean AUC across all bootstrap samples was 0.715 (95% CI:0.675 - 0.732).

The stability of the model’s performance was rigorously evaluated using an internal bootstrap validation with 1,000 replicates. The histogram distribution of the AUC values is approximately normal (**Figure 5**). The mean AUC across all bootstrap samples was 0.715, with 95% CI ranging from 0.675 to 0.732. The narrow confidence interval observed in the bootstrap samples reflects a high degree of stability in model performance. The bootstrap result is consistent with the AUC value of the original model of 0.722 (95% CI: 0.629–0.806). The findings indicate that the predictive performance of the aGVHD risk model based on IL-5, IL-8, and IL-10 exhibits good discrimination ability. The model can thus be considered a stable and reliable tool for supporting clinical assessment of aGVHD risk.

## 4 Discussion

In this retrospective study, the relationship between 12 cytokines, namely, IFN-α, IFN-γ, IL-1β, IL-2, IL-4, IL-5, IL-6, IL-8, IL-10, IL-12P70, IL-17A, and TNF-α, and the occurrence of aGVHD was investigated. IL-5, IL-8, and IL-10 levels were significantly different between patients who did and did not develop aGVHD. The levels of the three screened cytokines were elevated in patients with aGVHD compared with those in patients without aGVHD. The composite biomarker panel was more accurate than the individual biomarkers in predicting the development of aGVHD. Importantly, the composite panel provided significant predictive value beyond clinical factors. The internal bootstrap validation confirms that the aGVHD risk prediction model, based on the composite biomarker panel, possesses robust discriminatory power and demonstrates high stability, as evidenced by the narrow confidence intervals of the performance.

Despite the existence of numerous types of biomarkers, cytokine biomarkers are particularly favorable because of the extensive research conducted on cytokines in basic and clinical studies focusing on aGVHD (10). IL-5 and IL-10 are predominantly secreted by activated T helper type 2 (Th2) cells (18, 29). In patients with aGVHD, Th2 cells are considered to be regulatory T cells that inhibit T helper type 1 (Th1) cell responses, which induce aGVHD (29–31). Despite the paucity of studies on the analysis of the relationship between IL-5 levels and aGVHD, several studies have suggested that IL-5 may play an essential role in the onset of aGVHD (18, 31, 32). Moreover, elevated IL-5 levels are a hallmark of aGVHD-associated inflammation, indicating that the Th2 pathway is also activated in patients with aGVHD. IL-10 is a critical anti-inflammatory cytokine that effectively inhibits excessive inflammatory responses by negatively regulating the production and functions of multiple pro-inflammatory cytokines (33). IL-8, a neutrophil chemoattractant, exerts its primary biological function by recruiting and activating neutrophils, which play a role in acute inflammatory responses (34). IL-8 demonstrated only marginal significance in ROC analysis (adjusted P = 0.056) yet emerged as a robust independent predictor in Cox regression analysis. This suggests that the predictive value of IL-8 lies primarily in its ability to provide complementary information beyond the core signals from IL-5 and IL-10. The possible reason is that these cytokines interact with each other in the inflammatory pathway, and the independent contribution of IL-8 becomes fully evident after controlling for other factors. Thus, the combination of IL-5, IL-8, and IL-10 may more comprehensively reflect the underlying pathophysiological state and offer better diagnostic performance than any single biomarker alone. The findings suggest that the immunopathology of aGVHD not only involves the classic pro-inflammatory response but also includes complex regulatory and Th2-related pathways.

The composite biomarker panel (IL-5, IL-8, and IL-10) developed in this study was demonstrated to have a moderate discriminatory capacity [AUC: 0.722 (95% CI): 0.629 - 0.806) in predicting the occurrence of aGVHD. This performance is comparable to that of some of the published aGVHD biomarker models (35–37), confirming the feasibility of using peripheral blood cytokines for risk prediction. However, the performance remains lower than the superior predictive accuracy achieved using state-of-the-art, organ-specific biomarkers such as ST2 and REG3α, which directly reflect tissue damage in the gastrointestinal tract (38, 39). In contrast, the composite biomarker panel is indicative of systemic inflammation rather than organ-specific damage. However, unlike specialized tissue-damage biomarkers, in most clinical laboratories, the cytokines IL-5, IL-8, and IL-10 can be routinely measured, making the panel potentially easier and more cost-effective to implement, particularly in resource-limited settings. This accessibility indicates that the panel could be used not as a replacement for high-specificity markers, but as a practical component of a sequential diagnostic strategy. In such strategies, the panel could serve as an initial, high-sensitivity screening tool to identify a broader at-risk patient population. Having been identified, these individuals could then undergo subsequent, more definitive testing using higher-specificity (although often more expensive) biomarkers such as ST2, thereby optimizing resource allocation and focusing intensive diagnostic efforts where they are most needed.

Although established biomarkers such as ST2 and REG3α can make an invaluable contribution to assessing tissue-specific damage and prognosis in aGVHD, they primarily reflect the downstream consequences of injury. However, in this study, we aimed to profile upstream immunological drivers. The 12 selected cytokines capture dynamic signals throughout the entire pathological cascade, from initial inflammation to T-cell effector responses, thereby, providing a mechanism-based perspective for early risk assessment. This foundational work adopts these immune profiles as potential complementary partners to classical biomarkers, enabling a more comprehensive understanding of the onset and progression of aGVHD. Building on this basis, to further enhance predictive power, future steps should include prospective validation and the evaluation of combination models using established markers such as ST2 and REG3α.

The dynamic levels of cytokines such as IL-6, IL-8, and IL-10 are determined by a complex interplay of factors within the post-transplant milieu. In addition to the primary factors assessed in the present study, concurrent events such as infections, subclinical viral reactivations (e.g., CMV and EBV), and fluctuations in exposure to immunosuppressive drugs have been established to have a pronounced influence on these mediators. For example, even low-grade infections can serve as a non-specific inflammatory stimulus, potentially promoting an elevation in the baseline levels of IL-6 and IL-8. Similarly, variations in immunosuppression may contribute to altering the threshold for immune cell activation and cytokine release. In the present cohort, there were no significant differences between the experimental and control groups regarding the incidence of clinically documented infections (viral, bacterial, fungal) or the overall use of immunosuppressive drugs. However, although every effort was made to account for these factors in the analysis, residual confounding cannot be entirely excluded.

The major limitations of this study were the limited number of cytokines screened and the small sample size. Second, given the retrospective design, accurate grading of aGVHD severity and organ-specific involvement was not feasible, which accordingly precluded an analysis of associations between biomarker levels and disease grade or organ targets. Future prospective studies incorporating comprehensive clinical grading at diagnosis are thus needed to evaluate the utility of these cytokines for predicting disease severity and patterns of organ involvement. Third, in our analysis we focused on samples collected shortly prior to clinical diagnosis. However, although this design can effectively capture the immune profile at the time of disease onset, and is valuable for early diagnosis, it may miss the earliest predictive signals that emerge weeks in advance. Accordingly, to delineate the evolution of this cytokine signature and determine its long-term predictive utility, it will be necessary to undertake prospective studies incorporating frequent protocol-driven sampling, starting from the time of transplantation. Furthermore, the model lacks external validation, and its generalizability to other populations remains unconfirmed. Further validation through multicenter studies is needed to confirm the diagnostic potential of this composite biomarker panel.

## 5 Conclusions

This study demonstrated that a composite biomarker panel comprising IL-5, IL-8, and IL-10 may serve as an important biomarker for predicting the occurrence of aGVHD. However, further studies with large sample sizes are required to confirm the applicability of these candidate biomarkers in routine clinical practice. Future studies should incorporate additional cytokines to enhance the comprehensiveness of the research and facilitate an accurate prediction of aGVHD severity.

## Data Availability

The original contributions presented in the study are included in the article/**Supplementary Material**. Further inquiries can be directed to the corresponding author.
