# Supplementary material for: Panel of three cytokines predicts occurrence of aGVHD after allo-HSCT: a retrospective study
Source: Front Immunol. 2026 Jan 13;16:1719287. doi: 10.3389/fimmu.2025.1719287 (PMC12834795; doi:10.3389/fimmu.2025.1719287)
Supplement: Supplementary file 1 [file DataSheet1.docx]

**Supplementary**

**1. Cytokine levels between aGVHD and non-aGVHD patients**

**Table S1** **Cytokine levels between aGVHD and non-aGVHD patients**

| **Cytokines** | **non-aGVHD**  **(N = 90)** | **aGVHD**  **(N = 61)** | **P** | **adjusted P** |
| --- | --- | --- | --- | --- |
| IFN-α | 2.62 (1.38 - 4.56) | 2.69 (1.20 - 5.36) | 0.707 | 0.772 |
| IFN-γ | 3.70 (1.90 - 6.82) | 4.80 (2.84 - 7.67) | 0.094 | 0.227 |
| IL-1β | 1.86 (1.05 - 3.25) | 2.14 (1.31 - 3.59) | 0.163 | 0.327 |
| IL-2 | 2.04 (0.99 - 3.49) | 2.38 (1.10 - 4.19) | 0.191 | 0.328 |
| IL-4 | 2.38 (1.11 - 4.26) | 3.06 (1.11 - 4.07) | 0.542 | 0.722 |
| IL-5 | 1.91 (1.15 - 3.33) | 3.45 (1.76 - 5.62) | 0.002 | 0.009 |
| IL-6 | 13.19 (7.87 - 27.10) | 22.23 (9.94 - 44.23) | 0.019 | 0.058 |
| IL-8 | 19.13 (11.43 - 33.81) | 29.25 (16.71 - 53.77) | 0.003 | 0.012 |
| IL-10 | 7.27 (4.83 - 12.43) | 11.79 (7.14 - 20.17) | < 0.001 | 0.009 |
| IL-12P70 | 2.04 (1.01 - 3.64) | 2.12 (0.93 - 3.89) | 0.836 | 0.836 |
| IL-17A | 2.31 (1.19 - 7.68) | 2.80 (1.83 - 5.73) | 0.457 | 0.686 |
| TNF-α | 2.56 (1.55 - 4.01) | 2.62 (1.61 - 3.90) | 0.684 | 0.772 |

Note: Values were presented as median (IQR), with cytokine concentrations expressed in picograms per milliliter (pg/mL). P values were adjusted for multiple comparisons using the Benjamini-Hochberg false discovery rate method.

**
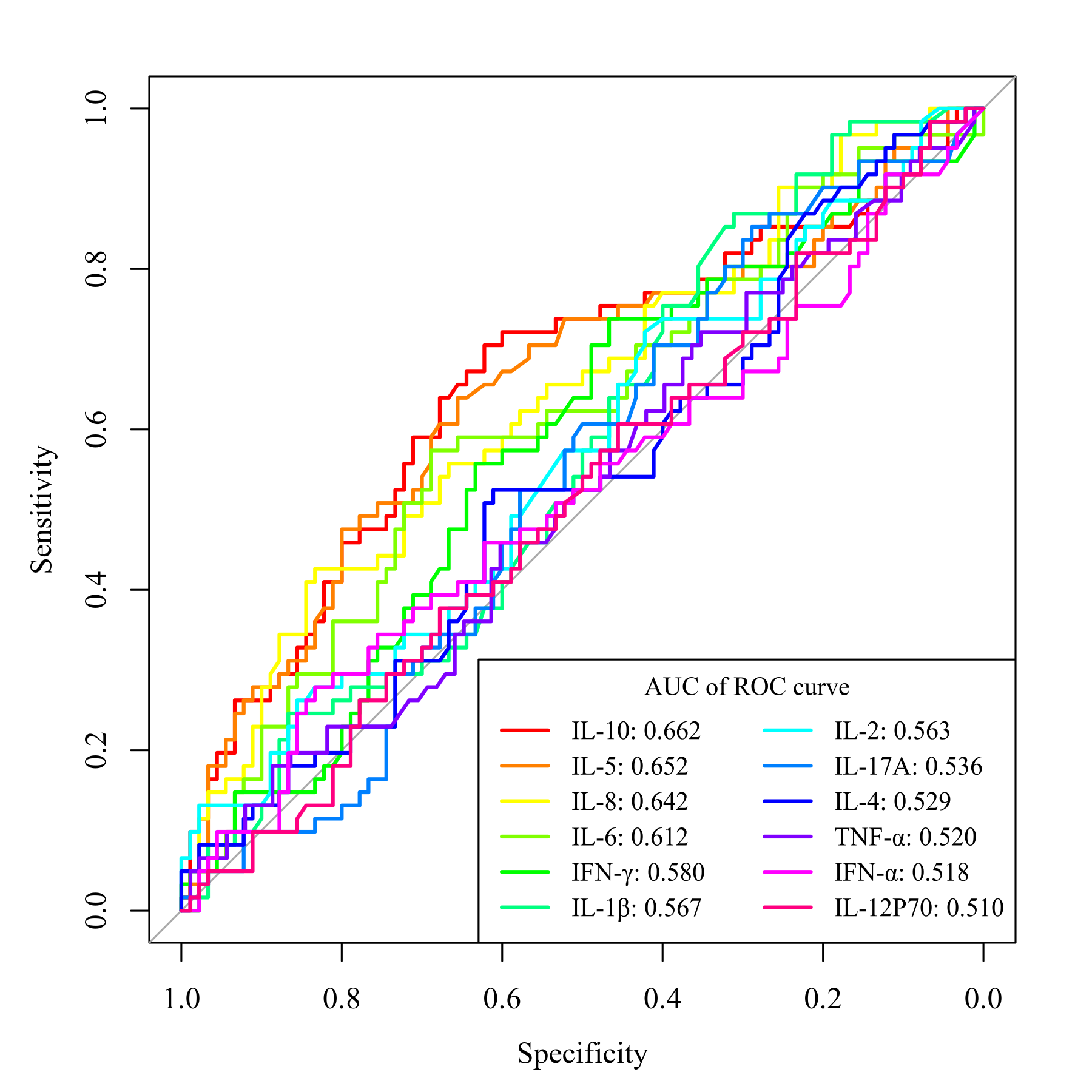
2. Performance of individual cytokines in predicting aGVHD**

**Fig. S1 Performance of individual cytokines in predicting aGVHD**

Receiver operating characteristic (ROC) curves depicting the predictive ability of 12 individual cytokines for aGVHD. The area under the curve (AUC) values for each cytokine are listed in Supplementary Table S2. The cytokine with the highest predictive value was IL-10 (AUC = 0.662 [95% CI: 0.566 - 0.752]).

**3. Predictive Performance of the 12 Cytokines for aGVHD**

**Table S2 Predictive Performance of the 12 Cytokines for aGVHD**

| **Cytokines** | **Cut-off Value (pg/mL)** | **Sensitivity (%)** | **Specificity (%)** | **AUC** | **P** | **adjusted P** |
| --- | --- | --- | --- | --- | --- | --- |
| IFN-α | 5.250 | 27.87 | 83.33 | 0.518 [95% CI: 0.469 - 0.617] | 0.159 | 0.211 |
| IFN-γ | 3.145 | 73.77 | 46.67 | 0.580 [95% CI: 0.492 - 0.672] | 0.036 | 0.086 |
| IL-1β | 1.115 | 86.89 | 31.11 | 0.567 [95% CI: 0.438 - 0.658] | 0.243 | 0.243 |
| IL-2 | 1.545 | 72.13 | 42.22 | 0.563 [95% CI: 0.464 - 0.661] | 0.126 | 0.211 |
| IL-4 | 2.875 | 52.46 | 61.11 | 0.529 [95% CI: 0.458 - 0.627] | 0.193 | 0.211 |
| IL-5 | 2.600 | 63.93 | 65.56 | 0.652 [95% CI: 0.562 - 0.750] | 0.001 | 0.006 |
| IL-6 | 18.880 | 57.38 | 68.89 | 0.612 [95% CI: 0.502 - 0.708] | 0.024 | 0.072 |
| IL-8 | 36.820 | 42.62 | 83.33 | 0.642 [95% CI: 0.542 - 0.733] | 0.014 | 0.056 |
| IL-10 | 8.615 | 70.49 | 62.22 | 0.662 [95% CI: 0.566 - 0.752] | <0.001 | <0.001 |
| IL-12P70 | 1.735 | 60.66 | 45.56 | 0.510 [95% CI: 0.472 - 0.616] | 0.182 | 0.211 |
| IL-17A | 1.330 | 85.25 | 28.89 | 0.536 [95% CI: 0.471 - 0.630] | 0.149 | 0.211 |
| TNF-α | 1.875 | 72.13 | 35.23 | 0.520 [95% CI: 0.469 - 0.615] | 0.163 | 0.211 |

Note: P values were adjusted for multiple comparisons using the Benjamini-Hochberg false discovery rate method.

**
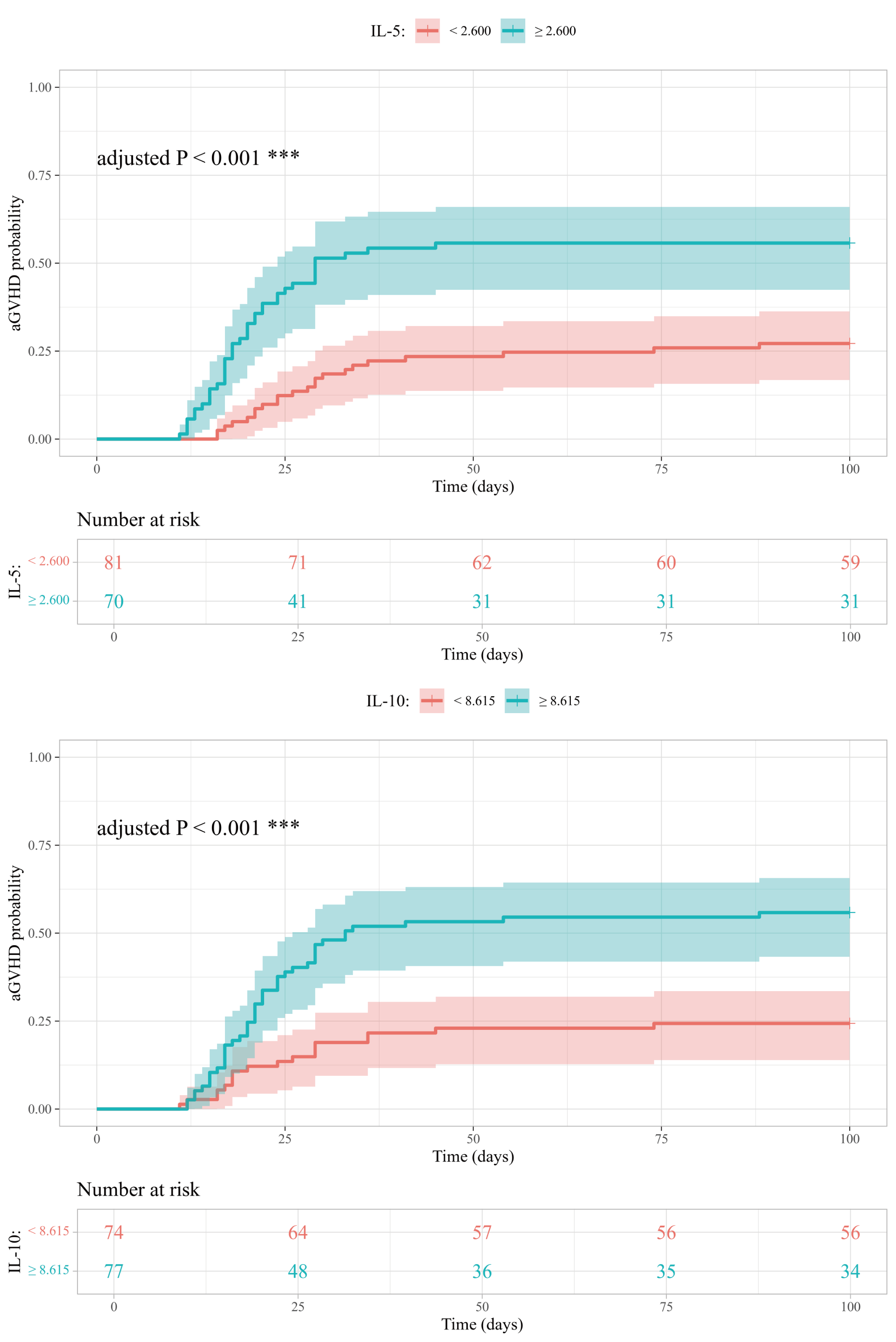
4. Cumulative Incidence of aGVHD Stratified by Cytokine Levels**

**Fig. S2 Cumulative Incidence of aGVHD Stratified by Cytokine Levels**

Kaplan–Meier curves illustrate the comparison of aGVHD incidence between high-level (green line) and low-level (red line) groups for the cytokines IL-5 and IL-10. The shaded areas represent the 95% confidence intervals. The number of patients at risk at each time point is shown in the corresponding table below the curves. Statistical significance was assessed using the log-rank test, and P values were adjusted for multiple comparisons using the Benjamini–Hochberg false discovery rate correction. (*, adjusted P < 0.05; **, adjusted P < 0.01; ***, adjusted P < 0.001).
